# Performance of Machine Learning Algorithms for Predicting Adverse Outcomes in Community-Acquired Pneumonia

**DOI:** 10.3389/fbioe.2022.903426

**Published:** 2022-06-29

**Authors:** Zhixiao Xu, Kun Guo, Weiwei Chu, Jingwen Lou, Chengshui Chen

**Affiliations:** ^1^ Department of Pulmonary and Critical Care Medicine, The First Affiliated Hospital of Wenzhou Medical University, Wenzhou, China; ^2^ The Interventional Pulmonary Key Laboratory of Zhejiang Province, Wenzhou, China

**Keywords:** machine learning, community-acquired pneumonia, CAP, adverse outcomes, XGBoost

## Abstract

**Background:** The ability to assess adverse outcomes in patients with community-acquired pneumonia (CAP) could improve clinical decision-making to enhance clinical practice, but the studies remain insufficient, and similarly, few machine learning (ML) models have been developed.

**Objective:** We aimed to explore the effectiveness of predicting adverse outcomes in CAP through ML models.

**Methods:** A total of 2,302 adults with CAP who were prospectively recruited between January 2012 and March 2015 across three cities in South America were extracted from DryadData. After a 70:30 training set: test set split of the data, nine ML algorithms were executed and their diagnostic accuracy was measured mainly by the area under the curve (AUC). The nine ML algorithms included decision trees, random forests, extreme gradient boosting (XGBoost), support vector machines, Naïve Bayes, K-nearest neighbors, ridge regression, logistic regression without regularization, and neural networks. The adverse outcomes included hospital admission, mortality, ICU admission, and one-year post-enrollment status.

**Results:** The XGBoost algorithm had the best performance in predicting hospital admission. Its AUC reached 0.921, and accuracy, precision, recall, and F1-score were better than those of other models. In the prediction of ICU admission, a model trained with the XGBoost algorithm showed the best performance with AUC 0.801. XGBoost algorithm also did a good job at predicting one-year post-enrollment status. The results of AUC, accuracy, precision, recall, and F1-score indicated the algorithm had high accuracy and precision. In addition, the best performance was seen by the neural network algorithm when predicting death (AUC 0.831).

**Conclusions:** ML algorithms, particularly the XGBoost algorithm, were feasible and effective in predicting adverse outcomes of CAP patients. The ML models based on available common clinical features had great potential to guide individual treatment and subsequent clinical decisions.

## 1 Introduction

Lower respiratory tract infections are a serious threat to human health, as evidenced by injuring 471.8 million people and causing 2.6 million deaths in 2017 alone (GBD 2017 Causes of Death Collaborators, 2018). Three cities in South America revealed the region’s high illness burden of adult community-acquired pneumonia (CAP), with an incidence rate ranging from 1.76 to 7.03 per 1,000 person-years (Lopardo et al., 2018). Obviously, CAP is still one of the most serious clinical and public health problems in the world, despite advances in technology and perspectives related to CAP (Aliberti et al., 2019). Physicians frequently apply various severity scoring systems to evaluate the prognosis of CAP patients, and the CURB-65 score is one of the preferred scoring systems, especially in predicting short-term mortality (Ito et al., 2017). However, research into the predictive variables of CAP remains a major issue in clinical practice. On the other hand, different projects use different prognostic factors and develop distinct prediction models for various CAP groups. Not surprisingly, multicenter investigations are also required.

Machine learning (ML) is a field of artificial intelligence and has many advantages. First, it enables predictions to be made using a range of approaches. Second, ML-based techniques could often be rigorously validated compared with that traditional statistical methods (Li R. et al., 2020). Third, model development is more data-driven and readily accommodates a large number of variables (Obermeyer and Emanuel, 2016), which allows the identification of previously unnoticed features to improve predictions (Woodman et al., 2021).

Most studies have reported the application of ML algorithms to novel coronavirus disease (COVID-19), but few to CAP (Mondal et al., 2021; Podder et al., 2021). Furthermore, many ML studies have focused on disease diagnosis; For example, ML could be applied to distinguish COVID-19 from CAP (Li L. et al., 2020; Podder et al., 2021; Qi et al., 2021), rather than on the prediction of outcomes. There are few studies that use machine learning to predict the dire outcomes in patients with CAP, especially one-year post-enrollment status. Therefore, in this study, we performed nine machine learning algorithms to evaluate the adverse outcomes of adult CAP patients.

The primary purpose was to develop feasible models based on multicenter data to predict specific adverse outcomes (including hospital admission, mortality, ICU admission, and one-year post-enrollment status), rather than to compare various learning algorithms as a research endpoint. Moreover, we hoped that the ML models based on available and common clinical-related feature variables developed in the study could be simple and accurate when medical practitioners assessed the prognosis of CAP patients.

## 2 Participants and Methods

### 2.1 Study Participants and Design

Through the PLOS ONE and DATADRYAD policy, we retrieved the raw data encompassing 2,302 adults with CAP who were prospectively recruited between January 2012 and March 2015 and performed a secondary analysis. (Lopardo et al., 2018) conducted a prospective cohort study of adult individuals across three cities in South America [General Roca (Argentina), Rivera (Uruguay), and Concepción (Paraguay)]. Participants were eligible for inclusion in the study if they 1) age ≥18 years old, 2) exited evidence of an acute lower respiratory infection, and 3) manifestation of radiologically confirmed pneumonia, defined as new or progressive pulmonary infiltrate(s) consistent with pneumonia detected on chest radiography or CT scan, and specific inclusion criteria can be found in the study by Lopardo et al. (Lopardo et al., 2018).

We did not infringe the rights of the authors when we used these data for secondary analysis, due to the original research has been ethically approved, and its authors have relinquished all copyright and related proprietary rights of these data. Therefore, no separate ethical approval was required for this study.

### 2.2 Outcome Definitions

Hospital admission, mortality, ICU admission, and one-year post-enrollment status were enrolled as outcomes in this study. One-year post-enrollment status indicated whether the CAP patients died at the one-year follow-up.

### 2.3 Candidate Predictors

34 clinically relevant variables were included in this study. The variables incorporated into our models included clinical signs (3 variables), clinical characteristics (6 variables), laboratory tests (8 variables), and comorbidities (17 variables).

### 2.4 Machine Learning Algorithms

The first stage in the data preprocessing step was to impute the missing values of continuous features such as clinical characteristics and laboratory testing using the feature’s mean value. Then, the data was divided into two sets at random in the ratio of 70:30, with the training set used to construct a model for each algorithm, and the test set used for a final evaluation of the accuracy of each algorithm. The prediction models were developed utilizing a systematic machine learning-based framework, and nine different machine learning algorithms included decision trees (DTs), random forests (RFs), eXtreme Gradient Boosting (XGBoost), support vector machines (SVM), naïve Bayes, K-nearest neighbors (KNN), ridge regression (logistic regression with L2 regularization), logistic regression without regularization, and neural networks (NNs). All features were employed, but no interactions or higher-order words were created.

A grid search of hyper-parameters was implemented for each algorithm to determine the optimal set of hyper-parameters for training data accuracy, and each grid search was carried out by using 10-fold cross-validation. The process was repeated 10 times, with each fold being used for one of the 10 training steps and for evaluating the model accuracy of the training data. Throughout the grid search, we employed the AUC as the accuracy statistic which is the most commonly used in clinical settings for comparison with other studies. However, for the completeness and accuracy of results, accuracy, precision, recall, and F1-score were also reported.

### 2.5 Statistical Analysis

All analyses were performed using Python version 3.8.8 and R version 4.1.1. The XGBoost algorithm was built with its own Python package, while other machine learning algorithms were developed using Python’s scikit-learn library.

## 3 Results

### 3.1 Characteristics of the Datasets Used for Machine Learning

A total of 2,302 patients were enrolled in the study. In the cohort, hospital admission occurred in 1,565 (68.0%) patients, death in 277 (12.0%) patients, ICU admission occurred in 343 (14.9%) patients, and one-year post-enrollment status in 1,621 (70.4%) patients. For age and clinical signs, there were no missing values. There were some missing values in laboratory tests and comorbidity among the variables utilized for machine learning. The patients’ characteristics were listed in Table 1 before the mean imputation for missing values. Moreover, the characteristics of the patients after mean imputation for missing values were also described according to four specific outcomes. According to hospital admission stratification, there were significant differences between the two groups for all features except for systolic blood pressure, leukocytes values, segmented neutrophils values, platelet values, creatinine, glucose, the history of intravenous drug use, the history of overcrowding, and the history of the received flu shot in the last 12 months (Table 2). And we could get similar results when stratifying based on other outcomes (Supplementary Tables S1–S3).

**TABLE 1:** Characteristics of the dataset used for machine learning before imputation.

	All	*n*
	*n* = 2,302	
Clinical signs		
Cough (%)		2,302
No	146 (6.34%)	
Yes	2,156 (93.7%)	
Dyspnea, tachypnoea, or hypoxemia (%)		2,302
No	543 (23.6%)	
Yes	1,759 (76.4%)	
Fever or hypothermia (%)		2,302
No	805 (35.0%)	
Yes	1,497 (65.0%)	
Clinical characteristics		
Age (year) (mean (SD))	63.3 (19.7)	2,302
Respiratory frequency (/min) (mean (SD))	28.0 (20.3)	2,100
Heart rate (/min) (mean (SD))	91.1 (16.5)	2,125
Systolic blood pressure (SBP) (mmHg) (mean (SD))	120 (24.5)	2,134
Diastolic blood pressure (DBP) (mmHg) (mean (SD))	73.6 (16.5)	2,133
CURB-65 (%)		1,449
Uncertain/Unknown	130 (8.97%)	
1	280 (19.3%)	
2	562 (38.8%)	
3	386 (26.6%)	
4	58 (4.00%)	
5	33 (2.28%)	
Laboratory tests		
Hematocrit values (%) (mean (SD))	37.4 (11.0)	1,790
Hemoglobin values (g/dl) (mean (SD))	12.6 (7.02)	1,765
Leukocytes values (10^9/L) (mean (SD))	14.6 (76.2)	1,811
Segmented neutrophils values (%) (mean (SD))	79.1 (12.8)	1,673
Platelet values (10^9/L) (mean (SD))	879 (23,412)	1,377
Creatinine (mg/dl) (mean (SD))	1.56 (4.55)	1,434
BUN (mg/dl) (mean (SD))	53.0 (38.8)	1,535
Glucose (mg/dl) (mean (SD))	137 (76.0)	1,377
Comorbidity		
Chronic obstructive pulmonary disease (COPD) (%)		2,302
Uncertain/unknown	59 (2.56%)	
No	1,898 (82.5%)	
Yes	345 (15.0%)	
Heart disease (%)		2,302
Uncertain/unknown	25 (1.09%)	
No	1,288 (56.0%)	
Yes	989 (43.0%)	
Diabetes (%)		2,302
Uncertain/unknown	16 (0.70%)	
No	1,924 (83.6%)	
Yes	362 (15.7%)	
Immunosuppression (%)		2,302
Uncertain/unknown	12 (0.52%)	
No	2,151 (93.4%)	
Yes	139 (6.04%)	
Malignancy (%)		2,302
Uncertain/unknown	12 (0.52%)	
No	2,171 (94.3%)	
Yes	119 (5.17%)	
Cerebrovascular disease (CBVD) (%)		2,302
Uncertain/unknown	15 (0.65%)	
No	2,126 (92.4%)	
Yes	161 (6.99%)	
Kidney disease (%)		2,302
Uncertain/unknown	15 (0.65%)	
No	2,130 (92.5%)	
Yes	157 (6.82%)	
Liver disease (%)		2,302
Uncertain/unknown	9 (0.39%)	
No	2,234 (97.0%)	
Yes	59 (2.56%)	
Intravenous drug use (%)		2,302
Uncertain/unknown	8 (0.35%)	
No	2,284 (99.2%)	
Yes	10 (0.43%)	
Alcoholism		2,302
Uncertain/unknown	30 (1.30%)	
No	2,134 (92.7%)	
Yes	138 (5.99%)	
Neurological psychiatric disorder (%)		2,302
Uncertain/unknown	33 (1.43%)	
No	1,927 (83.7%)	
Yes	342 (14.9%)	
Suspected aspiration (%)		2,302
Uncertain/unknown	20 (0.87%)	
No	2,223 (96.6%)	
Yes	59 (2.56%)	
Hospitalization due to CAP in previous year (%)		2,302
Uncertain/unknown	9 (0.39%)	
No	2,004 (87.1%)	
Yes	289 (12.6%)	
Overcrowding (%)		2,302
Uncertain/unknown	39 (1.69%)	
No	2,211 (96.0%)	
Yes	52 (2.26%)	
Smoking (%)		2,302
Uncertain/unknown	175 (7.60%)	
No	1,284 (55.8%)	
Yes	843 (36.6%)	
Received flu shot in the last 12 months (%)		2,302
Uncertain/unknown	34 (1.48%)	
No	1,559 (67.7%)	
Yes	709 (30.8%)	
Received antipneumococcic vaccine at any given time (%)		2,302
Uncertain/unknown	30 (1.30%)	
No	1,869 (81.2%)	
Yes	403 (17.5%)	
Outcomes		
Hospital admission (%)		2,302
Uncertain/unknown	2 (0.09%)	
No	735 (31.9%)	
Yes	1,565 (68.0%)	
Death (%)		2,302
Uncertain/unknown	21 (0.91%)	
No	2,004 (87.1%)	
Yes	277 (12.0%)	
ICU admission (%)		2,302
Uncertain/unknown	72 (3.13%)	
No	1,887 (82.0%)	
Yes	343 (14.9%)	
One-year post-enrollment status (%)		2,302
Uncertain/unknown	144 (6.26%)	
No	537 (23.3%)	
Yes	1,621 (70.4%)	

**TABLE 2:** Patient characteristics according to hospital admission stratification.

	Hospital admission		*p* overall
	No	Yes	
	*n* = 735	*n* = 1,565	
Cough (%)			0.001
No	28 (3.81%)	118 (7.54%)	
Yes	707 (96.2%)	1,447 (92.5%)	
Dyspnea, tachypnoea, or hypoxemia (%)			<0.001
No	250 (34.0%)	292 (18.7%)	
Yes	485 (66.0%)	1,273 (81.3%)	
Fever or hypothermia (%)			<0.001
No	205 (27.9%)	600 (38.3%)	
Yes	530 (72.1%)	965 (61.7%)	
Age (year) (mean (SD))	54.7 (19.6)	67.4 (18.4)	<0.001
Respiratory frequency (/min) (mean (SD))	29.6 (23.9)	27.3 (16.7)	0.019
Heart rate (/min) (mean (SD))	89.0 (12.1)	92.0 (17.3)	<0.001
SBP (mmHg) (mean (SD))	120 (18.2)	120 (25.7)	0.944
DBP (mmHg) (mean (SD))	77.0 (16.6)	72.0 (15.2)	<0.001
CURB-65 (mean (SD))	2.16 (0.36)	2.28 (0.80)	<0.001
Hematocrit values (%) (mean (SD))	38.1 (3.23)	37.1 (11.5)	0.001
Hemoglobin values (g/dl) (mean (SD))	13.2 (7.72)	12.4 (5.23)	0.005
Leukocytes values (10^9/L) (mean (SD))	13.5 (3.37)	15.1 (82.0)	0.425
Segmented neutrophils values (%) (mean (SD))	79.2 (5.72)	79.1 (12.6)	0.796
Platelet values (10^9/L) (mean (SD))	1,861 (32,030)	418 (296)	0.222
Creatinine (mg/dl) (mean (SD))	1.48 (0.54)	1.60 (4.34)	0.257
BUN (mg/dl) (mean (SD))	50.1 (15.0)	54.3 (37.0)	<0.001
Glucose (mg/dl) (mean (SD))	134 (30.5)	138 (68.1)	0.105
COPD (%)			<0.001
Uncertain/unknown	19 (2.59%)	39 (2.49%)	
No	645 (87.8%)	1,252 (80.0%)	
Yes	71 (9.66%)	274 (17.5%)	
Heart disease (%)			<0.001
Uncertain/unknown	2 (0.27%)	23 (1.47%)	
No	508 (69.1%)	780 (49.8%)	
Yes	225 (30.6%)	762 (48.7%)	
Diabetes (%)			<0.001
Uncertain/unknown	6 (0.82%)	10 (0.64%)	
No	662 (90.1%)	1,260 (80.5%)	
Yes	67 (9.12%)	295 (18.8%)	
Immunosuppression (%)			0.001
Uncertain/unknown	2 (0.27%)	10 (0.64%)	
No	707 (96.2%)	1,442 (92.1%)	
Yes	26 (3.54%)	113 (7.22%)	
Malignancy (%)			<0.001
Uncertain/unknown	5 (0.68%)	7 (0.45%)	
No	717 (97.6%)	1,452 (92.8%)	
Yes	13 (1.77%)	106 (6.77%)	
CBVD (%)			<0.001
Uncertain/unknown	3 (0.41%)	12 (0.77%)	
No	712 (96.9%)	1,412 (90.2%)	
Yes	20 (2.72%)	141 (9.01%)	
Kidney disease (%)			<0.001
Uncertain/unknown	6 (0.82%)	9 (0.58%)	
No	708 (96.3%)	1,421 (90.8%)	
Yes	21 (2.86%)	135 (8.63%)	
Liver disease (%)			0.009
Uncertain/unknown	5 (0.68%)	4 (0.26%)	
No	720 (98.0%)	1,512 (96.6%)	
Yes	10 (1.36%)	49 (3.13%)	
Intravenous drug use (%)			0.252
Uncertain/unknown	4 (0.54%)	3 (0.19%)	
No	729 (99.2%)	1,554 (99.3%)	
Yes	2 (0.27%)	8 (0.51%)	
Alcoholism (%)			0.001
Uncertain/unknown	7 (0.95%)	23 (1.47%)	
No	703 (95.6%)	1,429 (91.3%)	
Yes	25 (3.40%)	113 (7.22%)	
Neurological psychiatric disorder (%)			<0.001
Uncertain/unknown	5 (0.68%)	28 (1.79%)	
No	699 (95.1%)	1,227 (78.4%)	
Yes	31 (4.22%)	310 (19.8%)	
Suspected aspiration (%)			<0.001
Uncertain/unknown	4 (0.54%)	16 (1.02%)	
No	729 (99.2%)	1,492 (95.3%)	
Yes	2 (0.27%)	57 (3.64%)	
Hospitalization due to CAP in previous year (%)			<0.001
Uncertain/unknown	3 (0.41%)	6 (0.38%)	
No	685 (93.2%)	1,318 (84.2%)	
Yes	47 (6.39%)	241 (15.4%)	
Overcrowding (%)			0.797
Uncertain/unknown	14 (1.90%)	24 (1.53%)	
No	705 (95.9%)	1,505 (96.2%)	
Yes	16 (2.18%)	36 (2.30%)	
Smoking (%)			0.009
Uncertain/unknown	39 (5.31%)	136 (8.69%)	
No	432 (58.8%)	850 (54.3%)	
Yes	264 (35.9%)	579 (37.0%)	
Received flu shot in the last 12 months (%)			0.092
Uncertain/unknown	5 (0.68%)	29 (1.85%)	
No	504 (68.6%)	1,054 (67.3%)	
Yes	226 (30.7%)	482 (30.8%)	
Received antipneumococcic vaccine at any given time (%)			<0.001
Uncertain/unknown	4 (0.54%)	26 (1.66%)	
No	633 (86.1%)	1,234 (78.8%)	
Yes	98 (13.3%)	305 (19.5%)	

### 3.2 Performance of the Machine Learning Model

#### 3.2.1 Outcome 1: Hospital Admission

**FIGURE 1:**
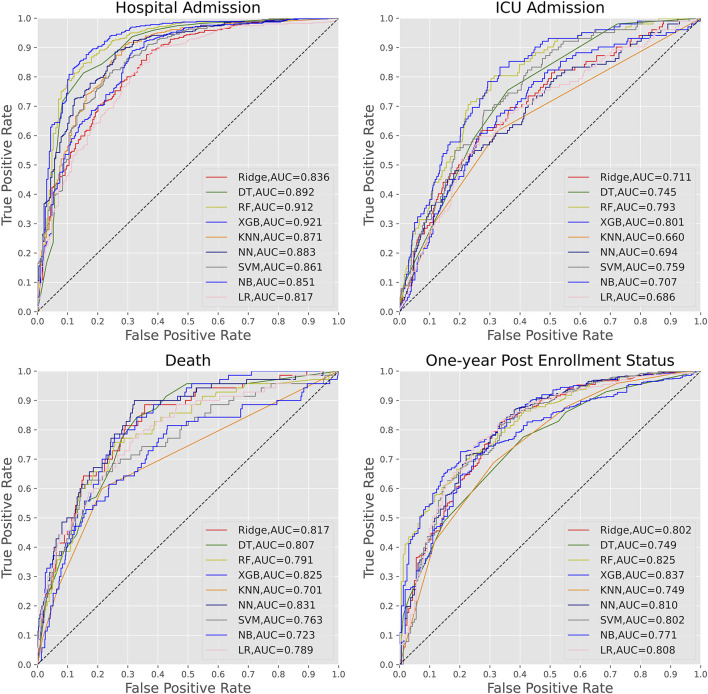
Test accuracy of the nine machine learning algorithms for the prediction of hospital admission, ICU admission, death and one-year enrollment status.

The test accuracy results were shown in Table 3 and Figure 1. The AUC for XGBoost (AUC = 0.921) was considerably higher than other ML algorithms. The results of the accuracy score, precision score, recall score, and F1-score for the nine ML algorithms were also provided. Overall, the best-performing ML algorithm was XGBoost. As with the AUC, values for accuracy score and precision score were comparable across the various models with a range of 0.716 (Naïve Bayes) to 0.877 (XGBoost) for accuracy score, a range of 0.816 (Ridge) to 0.9 (KNN) for the precision score, a range of 0.662 (Naïve Bayes) to 0.957 (random forest) for recall score and a range of 0.801 (KNN) to 0.911 (XGBoost) for F1-score.

**TABLE 3:** Diagnostic accuracy for the nine machine learning algorithms with the test dataset for the prediction of hospital admission and ICU admission.

	Hospital admission					ICU admission				
	Accuracy score	Precision score	Recall score	F1-score	AUC	Accuracy score	Precision score	Recall score	F1-score	AUC
Ridge	0.799	0.816	0.902	0.857	0.836	0.846	0.444	0.039	0.072	0.711
DT	0.854	0.857	0.937	0.895	0.892	0.848	0.500	0.098	0.164	0.745
RF	0.857	0.848	0.957	0.899	0.912	0.846	0.455	0.049	0.088	0.793
XGB	0.877	0.879	0.946	0.911	0.921	0.846	0.478	0.108	0.176	0.801
KNN	0.761	0.900	0.722	0.801	0.871	0.854	0.667	0.078	0.140	0.660
NN	0.832	0.856	0.900	0.877	0.883	0.839	0.312	0.049	0.085	0.694
SVM	0.810	0.833	0.896	0.863	0.861	0.854	0.833	0.049	0.093	0.759
NB	0.716	0.884	0.662	0.757	0.851	0.205	0.157	0.961	0.269	0.707
LR	0.778	0.822	0.852	0.837	0.817	0.831	0.359	0.137	0.199	0.686

DT, KNN, LR, NB, NN, RF, Ridge; SVM, and XGB represented decision tree, K-nearest neighbors, logistic regression without penalization, Naive Bayes, neural network, random forest, ridge regression, support vector machine, and eXtreme gradient boosting respectively.

#### 3.2.2 Outcome 2: ICU Admission

Although machine learning algorithms did not perform as well in the prediction of ICU admission as they did in the prediction of other outcomes, there were still the most, but not all, of the ML algorithms with AUC values > 0.7 (Table 3 and Figure 1). Furthermore, the AUC for XGBoost (AUC = 0.801) was considerably higher than other ML algorithms. Unfortunately, the recall scores and F1-score did not farewell.

#### 3.2.3 Outcome 3: Clinical Evolution (Death)

**FIGURE 2:**
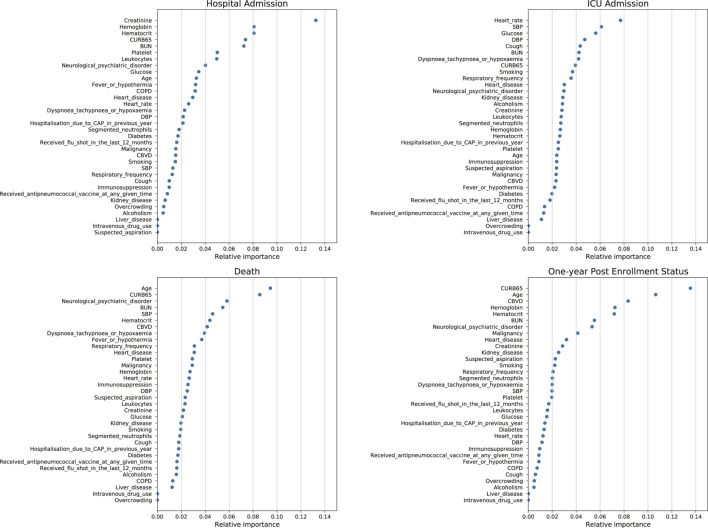
Feature importance plot for the eXtreme gradient boosting algorithm using test data for the prediction of hospital admission, ICU admission, death and one-year post-enrollment status.

For the prediction of death, the neural network had a much higher AUC than other machine learning methods (AUC = 0.831) (Table 4 and Figure 2). Table 4 showed that the precision, recall, and F1-score were all rather low, although the model had a high accuracy score of 0.893. We also noticed that XGBoost displayed the second highest AUC of 0.825.

**TABLE 4:** Diagnostic accuracy for the nine machine learning algorithms with the test dataset for the prediction of death and one-year post-enrollment status.

	Death					One-year post-enrollment status				
	Accuracy score	Precision score	Recall score	F1-score	AUC	Accuracy score	Precision score	Recall score	F1-score	AUC
Ridge	0.892	0.447	0.243	0.315	0.817	0.802	0.814	0.955	0.879	0.802
DT	0.876	0.368	0.300	0.331	0.807	0.775	0.801	0.930	0.861	0.749
RF	0.899	0.524	0.157	0.242	0.791	0.802	0.819	0.944	0.877	0.825
XGB	0.896	0.480	0.171	0.253	0.825	0.816	0.844	0.926	0.883	0.837
KNN	0.892	0.375	0.086	0.140	0.701	0.787	0.798	0.959	0.871	0.749
NN	0.893	0.465	0.286	0.354	0.831	0.810	0.843	0.918	0.879	0.810
SVM	0.895	0.469	0.214	0.294	0.763	0.804	0.827	0.934	0.877	0.802
NB	0.705	0.203	0.643	0.308	0.723	0.736	0.858	0.775	0.815	0.771
LR	0.896	0.488	0.286	0.360	0.789	0.804	0.842	0.909	0.874	0.808

#### 3.2.4 Outcome 4: One-Year Post-Enrollment Status (Death)

Overall, the nine machine learning algorithms did a good job at predicting one-year post-enrollment status. Similarly, the AUC for XGBoost (AUC = 0.837) was much higher than that of other machine learning algorithms. Except for the AUC values, the results of accuracy, precision, recall, and F1-score also indicated that the nine ML algorithms had high accuracy and precision, with values for accuracy and precision being comparable across the various models with a range of 0.736 (Naïve Bayes) to 0.816 (XGBoost) for accuracy score, a range of 0.798 (KNN) to 0.858 (Naïve Bayes) for the precision score, a range of 0.815 (Naïve Bayes) to 0.959 (KNN) for recall and a range of 0.815 (Naïve Bayes) to 0.883 (XGBoost) for F1-score.

### 3.3 Feature Importance and Distributions

Given the high accuracy and precision of the XGBoost for the prediction of all four outcomes, the feature importance plot of the XGBoost algorithm for the test dataset is also shown. For the prediction of hospital admission, creatinine had the highest feature importance. For the prediction of ICU admission, heart rate had the highest feature importance. For the prediction of death, age, and CURB-65 score had the highest feature importance. For the prediction of one-year post-enrollment status, age and CURB-65 score had the highest feature importance.

## 4 Discussion

Machine learning has become increasingly applied in medicine because of its computational power and the availability of massive new datasets. Most of the current research about machine learning focuses on distinguishing CAP from other diseases, such as COVID-19 (Li L. et al., 2020), while studies predicting the outcomes of pneumonia based on commonly used clinical test data are limited. In this study, we applied a systematic machine learning framework to data from 2,302 adult CAP patients in three cities in South America to develop and test predictive models that could potentially be used in clinical settings to assist with risk management. And a grid search of the hyperparameter space and 10-fold cross-validation for each algorithm was performed to ensure the reliability of the results.

As a result, our models offered very high accuracy in detecting the adverse outcomes of CAP patients. This study predicted multiple adverse of pneumonia, which was innovative noticeably. In detail, the test data accuracy using the clinical signs and clinical characteristics and laboratory tests and comorbidities reached an AUC of 0.921 using the XGBoost algorithm for the prediction of hospital admission, an AUC of 0.831 using the neural network algorithm for the prediction of death, an AUC of 0.801 using XGBoost algorithm for the prediction of ICU admission, and an AUC of 0.837 using XGBoost algorithm for the prediction of one-year post-enrollment status.

To make the models implementable in clinical practice, the models were trained using variables commonly available to medical practitioners. We included four adverse outcomes and used 34 variables to generate the predictive models for adverse outcomes. Then, the medical team could make a judgment and take immediate action. Potentially important implications of the development of predictive models for optimizing post-hospital monitoring and the quality of care (Brajer et al., 2020; D'Ascenzo et al., 2021).

At present, only a few studies have reported the application of machine learning in pneumonia-related studies. And several studies have employed machine learning methods to predict mortality from pneumonia (Cooper et al., 1997; Wiemken et al., 2017; Kang et al., 2020), while other adverse outcomes have received less attention, especially one-year post-enrollment status. Feng et al. built a three-layer fully connected neural network to classify the prognosis of CAP patients with high accuracy and good generalizability by using ML techniques to predict CAP mortality (Feng et al., 2021). The proposed ML-based models including the CURB-65 score could accurately predict the death within 30 days or initial admission to the ICU from the emergency department with an AUC of 0.844 (Kang et al., 2020). Cooper et al. constructed 11 statistical and machine learning models that predict dire outcomes for CAP patients (such as mortality or severe clinical complications) and discovered an innovative neural network learning method that induced a model using Multitask and Learning along with Rank-prop learning (MTLR) with the largest ROC area (Cooper et al., 2005).

Models that highlight vital signs and laboratory tests may be more valid in alerting healthcare workers to potentially modifiable organ failure than models that rely heavily on comorbidities and demographic characteristics (Jones et al., 2021). Based on this spirit, our study established the accuracy of computerized prediction and revealed that the XGBoost algorithm was reliable. Moreover, the XGBoost algorithm is one of the powerful ML algorithms that may also align more closely with human thought (Fong et al., 2018). We found that many mainstream prognostic features still held important weight. The feature importance plots for the XGBoost algorithm for the prediction of admission, death, and one-year post-enrollment status showed the importance of CURB-65, which all ranked in the top 5. The CURB-65 score is one of the most commonly used predictive models for the classification of patients suffering from fever, dyspnea, and upper/lower respiratory symptoms (e.g., coughing). CURB-65 is much less computationally time-consuming; however, it has a disadvantage in that it contains only five variables (Buising et al., 2006; Capelastegui et al., 2006; Man et al., 2007). But in the cohort of elderly patients hospitalized for pneumonia in Geneva University Hospitals, the CURB-65 score was not predictive of one-year mortality (Malézieux-Picard et al., 2021).

It is incorrect to conclude that a model is a prediction of events. In fact, a model is a summarization of the collective clinical experience of events that happen in patients with similar clinical characteristics (Weick and Roberts, 1993). The value of prediction is that it places the clinical features of a new patient in the context of that clinical experience, providing a common basis for communication among clinicians, especially for those unfamiliar with each other (Jones et al., 2021). Credible models are essential to providing reliable, efficient, and equitable health care, and the models we have built are paving the way for that process.

This study has several limitations. First, despite the fact that patient data were obtained prospectively, missing values were unavoidable, and these missing values would skew the study’s conclusions. Second, in comparison with standard statistical models, ML methods have been found to generally require bigger data sets and, in particular, a higher number of events before the stable measures of prediction performance could be obtained (van der Ploeg et al., 2014). Despite this limitation, we still achieved moderate to high validation accuracy using data sets with a relatively limited number of events. Third, although 34 variables were included in this study, they still did not cover very comprehensively. Moreover, four adverse outcomes were investigated in the present study, but there are still many adverse outcomes that occur in clinical practice that have not been addressed.

The prospective validation of operational data is a critical first step in assessing the real-world performance of machine learning models. In the future, we intend to undertake prospective multicenter large-sample research to further prove the model’s utility. Furthermore, more clinical features including imaging features and more adverse outcomes should be included.

## 5 Conclusion

In the study, we have developed and tested the machine learning-based model to predict hospital admission, mortality, ICU admission, and one-year post-enrollment status in CAP patients. The results revealed that the ML algorithms (especially the XGBoost algorithm) were feasible and effective. There is potential to improve clinical practice if ML models based on available and common clinical-related feature variables are incorporated into future clinical decision aids when assessing the prognosis of patients with CAP. Furthermore, prospective multicenter large-sample research including more clinical features is required.

## Data Availability

Data acquisition from DryadData database (https://doi.org/10.5061/dryad.r282vk6). And other contributions presented in the study are included in the article/Supplementary Material; further inquiries can be directed to the corresponding author.
