# Supplementary material for: Performance of Machine Learning Algorithms for Predicting Adverse Outcomes in Community-Acquired Pneumonia
Source: Front Bioeng Biotechnol. 2022 Jun 29;10:903426. doi: 10.3389/fbioe.2022.903426 (PMC9278327; doi:10.3389/fbioe.2022.903426)
Supplement: Supplementary file 1 [file Table1.DOCX]

Table S1. Patient characteristics according to ICU admission stratification.

|  | ICU admission | | p.overall |
| --- | --- | --- | --- |
|  | No | Yes |  |
|  | N=1887 | N=343 |  |
| Cough (%) |  |  | <0.001 |
| No | 99 (5.25%) | 44 (12.8%) |  |
| Yes | 1788 (94.8%) | 299 (87.2%) |  |
| Dyspnea, tachypnoea or hypoxemia (%) |  |  | <0.001 |
| No | 481 (25.5%) | 41 (12.0%) |  |
| Yes | 1406 (74.5%) | 302 (88.0%) |  |
| Fever or hypothermia (%) |  |  | 0.021 |
| No | 645 (34.2%) | 140 (40.8%) |  |
| Yes | 1242 (65.8%) | 203 (59.2%) |  |
| Age (year) (mean (SD)) | 62.9 (19.7) | 67.6 (18.1) | <0.001 |
| Respiratory frequency (/min) (mean (SD)) | 28.1 (20.3) | 28.8 (15.5) | 0.517 |
| Heart rate (/min) (mean (SD)) | 89.6 (14.5) | 99.0 (20.8) | <0.001 |
| SBP (mmHg) (mean (SD)) | 120 (21.8) | 117 (32.6) | 0.077 |
| DBP (mmHg) (mean (SD)) | 74.2 (15.4) | 70.6 (18.8) | 0.001 |
| CURB-65 (mean (SD)) | 2.20 (0.62) | 2.52 (1.00) | <0.001 |
| Hematocrit values (%) (mean (SD)) | 37.3 (7.00) | 37.6 (18.9) | 0.831 |
| Hemoglobin values (g/dl) (mean (SD)) | 12.7 (6.31) | 12.4 (5.85) | 0.341 |
| Leukocytes values (10^9/L) (mean (SD)) | 13.2 (7.17) | 22.7 (174) | 0.315 |
| Segmented neutrophils values (%) (mean (SD)) | 79.3 (10.5) | 78.0 (13.4) | 0.08 |
| Platelet values (10^9/L) (mean (SD)) | 975 (19996) | 418 (310) | 0.227 |
| Creatinine (mg/dL) (mean (SD)) | 1.54 (3.81) | 1.68 (2.60) | 0.375 |
| BUN (mg/dL) (mean (SD)) | 51.2 (28.1) | 62.2 (46.0) | <0.001 |
| Glucose (mg/dL) (mean (SD)) | 136 (56.9) | 141 (71.5) | 0.16 |
| COPD (%) |  |  | 0.629 |
| Uncertain/unknown | 49 (2.60%) | 9 (2.62%) |  |
| No | 1557 (82.5%) | 276 (80.5%) |  |
| Yes | 281 (14.9%) | 58 (16.9%) |  |
| Heart disease (%) |  |  | <0.001 |
| Uncertain/unknown | 23 (1.22%) | 2 (0.58%) |  |
| No | 1081 (57.3%) | 160 (46.6%) |  |
| Yes | 783 (41.5%) | 181 (52.8%) |  |
| Diabetes (%) |  |  | 0.192 |
| Uncertain/unknown | 12 (0.64%) | 4 (1.17%) |  |
| No | 1581 (83.8%) | 276 (80.5%) |  |
| Yes | 294 (15.6%) | 63 (18.4%) |  |
| Immunosuppression (%) |  |  | 0.132 |
| Uncertain/unknown | 11 (0.58%) | 1 (0.29%) |  |
| No | 1768 (93.7%) | 313 (91.3%) |  |
| Yes | 108 (5.72%) | 29 (8.45%) |  |
| Malignancy (%) |  |  | 0.283 |
| Uncertain/unknown | 12 (0.64%) | 0 (0.00%) |  |
| No | 1780 (94.3%) | 322 (93.9%) |  |
| Yes | 95 (5.03%) | 21 (6.12%) |  |
| CBVD (%) |  |  | 0.043 |
| Uncertain/unknown | 14 (0.74%) | 1 (0.29%) |  |
| No | 1750 (92.7%) | 307 (89.5%) |  |
| Yes | 123 (6.52%) | 35 (10.2%) |  |
| Kidney disease (%) |  |  | 0.008 |
| Uncertain/unknown | 9 (0.48%) | 4 (1.17%) |  |
| No | 1760 (93.3%) | 304 (88.6%) |  |
| Yes | 118 (6.25%) | 35 (10.2%) |  |
| Liver disease (%) |  |  | 0.325 |
| Uncertain/unknown | 7 (0.37%) | 2 (0.58%) |  |
| No | 1835 (97.2%) | 329 (95.9%) |  |
| Yes | 45 (2.38%) | 12 (3.50%) |  |
| Intravenous drug use (%) |  |  | 0.647 |
| Uncertain/unknown | 7 (0.37%) | 0 (0.00%) |  |
| No | 1872 (99.2%) | 341 (99.4%) |  |
| Yes | 8 (0.42%) | 2 (0.58%) |  |
| Alcoholism (%) |  |  | 0.017 |
| Uncertain/unknown | 27 (1.43%) | 3 (0.87%) |  |
| No | 1755 (93.0%) | 307 (89.5%) |  |
| Yes | 105 (5.56%) | 33 (9.62%) |  |
| Neurological psychiatric disorder (%) |  |  | <0.001 |
| Uncertain/unknown | 27 (1.43%) | 6 (1.75%) |  |
| No | 1597 (84.6%) | 261 (76.1%) |  |
| Yes | 263 (13.9%) | 76 (22.2%) |  |
| Suspected aspiration (%) |  |  | <0.001 |
| Uncertain/unknown | 20 (1.06%) | 0 (0.00%) |  |
| No | 1828 (96.9%) | 323 (94.2%) |  |
| Yes | 39 (2.07%) | 20 (5.83%) |  |
| Hospitalization due to CAP in previous year (%) |  |  | 0.049 |
| Uncertain/unknown | 9 (0.48%) | 0 (0.00%) |  |
| No | 1651 (87.5%) | 287 (83.7%) |  |
| Yes | 227 (12.0%) | 56 (16.3%) |  |
| Overcrowding (%) |  |  | 0.149 |
| Uncertain/unknown | 30 (1.59%) | 6 (1.75%) |  |
| No | 1808 (95.8%) | 334 (97.4%) |  |
| Yes | 49 (2.60%) | 3 (0.87%) |  |
| Smoking (%) |  |  | <0.001 |
| Uncertain/unknown | 120 (6.36%) | 44 (12.8%) |  |
| No | 1056 (56.0%) | 195 (56.9%) |  |
| Yes | 711 (37.7%) | 104 (30.3%) |  |
| Received flu shot in the last 12 months (%) |  |  | 0.049 |
| Uncertain/unknown | 24 (1.27%) | 10 (2.92%) |  |
| No | 1289 (68.3%) | 222 (64.7%) |  |
| Yes | 574 (30.4%) | 111 (32.4%) |  |
| Received antipneumococcic vaccine at any given time (%) |  |  | 0.03 |
| Uncertain/unknown | 21 (1.11%) | 9 (2.62%) |  |
| No | 1545 (81.9%) | 265 (77.3%) |  |
| Yes | 321 (17.0%) | 69 (20.1%) |  |
