# Supplementary material for: Performance of Machine Learning Algorithms for Predicting Adverse Outcomes in Community-Acquired Pneumonia
Source: Front Bioeng Biotechnol. 2022 Jun 29;10:903426. doi: 10.3389/fbioe.2022.903426 (PMC9278327; doi:10.3389/fbioe.2022.903426)
Supplement: Supplementary file 2 [file Table2.DOCX]

Table S2. Patient characteristics according to death stratification.

|  | Death | | p.overall |
| --- | --- | --- | --- |
|  | No | Yes |  |
|  | N=2004 | N=277 |  |
| Cough (%) |  |  | 0.619 |
| No | 125 (6.24%) | 20 (7.22%) |  |
| Yes | 1879 (93.8%) | 257 (92.8%) |  |
| Dyspnea, tachypnoea or hypoxemia (%) |  |  | <0.001 |
| No | 508 (25.3%) | 24 (8.66%) |  |
| Yes | 1496 (74.7%) | 253 (91.3%) |  |
| Fever or hypothermia (%) |  |  | <0.001 |
| No | 660 (32.9%) | 138 (49.8%) |  |
| Yes | 1344 (67.1%) | 139 (50.2%) |  |
| Age (year) (mean (SD)) | 61.5 (19.5) | 76.7 (15.3) | <0.001 |
| Respiratory frequency (/min) (mean (SD)) | 27.9 (19.7) | 29.6 (16.8) | 0.112 |
| Heart rate (/min) (mean (SD)) | 90.4 (15.3) | 96.1 (19.3) | <0.001 |
| SBP (mmHg) (mean (SD)) | 121 (22.6) | 112 (28.4) | <0.001 |
| DBP (mmHg) (mean (SD)) | 74.4 (15.7) | 68.1 (16.2) | <0.001 |
| CURB-65 (mean (SD)) | 2.18 (0.62) | 2.77 (0.94) | <0.001 |
| Hematocrit values (%) (mean (SD)) | 37.9 (9.97) | 34.0 (6.81) | <0.001 |
| Hemoglobin values (g/dl) (mean (SD)) | 12.8 (6.12) | 11.6 (6.49) | 0.005 |
| Leukocytes values (10^9/L) (mean (SD)) | 14.8 (72.4) | 13.3 (9.30) | 0.363 |
| Segmented neutrophils values (%) (mean (SD)) | 79.0 (10.6) | 80.0 (12.7) | 0.228 |
| Platelet values (10^9/L) (mean (SD)) | 956 (19403) | 396 (303) | 0.197 |
| Creatinine (mg/dL) (mean (SD)) | 1.56 (3.82) | 1.64 (1.27) | 0.497 |
| BUN (mg/dL) (mean (SD)) | 50.5 (27.6) | 71.1 (49.2) | <0.001 |
| Glucose (mg/dL) (mean (SD)) | 137 (58.2) | 138 (63.4) | 0.798 |
| COPD (%) |  |  | 0.154 |
| Uncertain/unknown | 56 (2.79%) | 3 (1.08%) |  |
| No | 1642 (81.9%) | 237 (85.6%) |  |
| Yes | 306 (15.3%) | 37 (13.4%) |  |
| Heart disease (%) |  |  | <0.001 |
| Uncertain/unknown | 24 (1.20%) | 1 (0.36%) |  |
| No | 1158 (57.8%) | 115 (41.5%) |  |
| Yes | 822 (41.0%) | 161 (58.1%) |  |
| Diabetes (%) |  |  | 0.557 |
| Uncertain/unknown | 14 (0.70%) | 2 (0.72%) |  |
| No | 1681 (83.9%) | 226 (81.6%) |  |
| Yes | 309 (15.4%) | 49 (17.7%) |  |
| Immunosuppression (%) |  |  | 0.055 |
| Uncertain/unknown | 8 (0.40%) | 4 (1.44%) |  |
| No | 1877 (93.7%) | 253 (91.3%) |  |
| Yes | 119 (5.94%) | 20 (7.22%) |  |
| Malignancy (%) |  |  | <0.001 |
| Uncertain/unknown | 11 (0.55%) | 1 (0.36%) |  |
| No | 1916 (95.6%) | 236 (85.2%) |  |
| Yes | 77 (3.84%) | 40 (14.4%) |  |
| CBVD (%) |  |  | <0.001 |
| Uncertain/unknown | 11 (0.55%) | 4 (1.44%) |  |
| No | 1878 (93.7%) | 227 (81.9%) |  |
| Yes | 115 (5.74%) | 46 (16.6%) |  |
| Kidney disease (%) |  |  | <0.001 |
| Uncertain/unknown | 14 (0.70%) | 1 (0.36%) |  |
| No | 1874 (93.5%) | 236 (85.2%) |  |
| Yes | 116 (5.79%) | 40 (14.4%) |  |
| Liver disease (%) |  |  | 0.514 |
| Uncertain/unknown | 9 (0.45%) | 0 (0.00%) |  |
| No | 1946 (97.1%) | 268 (96.8%) |  |
| Yes | 49 (2.45%) | 9 (3.25%) |  |
| Intravenous drug use (%) |  |  | 0.42 |
| Uncertain/unknown | 8 (0.40%) | 0 (0.00%) |  |
| No | 1988 (99.2%) | 275 (99.3%) |  |
| Yes | 8 (0.40%) | 2 (0.72%) |  |
| Alcoholism (%) |  |  | 0.016 |
| Uncertain/unknown | 28 (1.40%) | 1 (0.36%) |  |
| No | 1867 (93.2%) | 250 (90.3%) |  |
| Yes | 109 (5.44%) | 26 (9.39%) |  |
| Neurological psychiatric disorder (%) |  |  | <0.001 |
| Uncertain/unknown | 24 (1.20%) | 9 (3.25%) |  |
| No | 1727 (86.2%) | 180 (65.0%) |  |
| Yes | 253 (12.6%) | 88 (31.8%) |  |
| Suspected aspiration (%) |  |  | <0.001 |
| Uncertain/unknown | 12 (0.60%) | 8 (2.89%) |  |
| No | 1953 (97.5%) | 249 (89.9%) |  |
| Yes | 39 (1.95%) | 20 (7.22%) |  |
| Hospitalization due to CAP in previous year (%) |  |  | <0.001 |
| Uncertain/unknown | 6 (0.30%) | 3 (1.08%) |  |
| No | 1769 (88.3%) | 216 (78.0%) |  |
| Yes | 229 (11.4%) | 58 (20.9%) |  |
| Overcrowding (%) |  |  | 0.374 |
| Uncertain/unknown | 30 (1.50%) | 7 (2.53%) |  |
| No | 1928 (96.2%) | 265 (95.7%) |  |
| Yes | 46 (2.30%) | 5 (1.81%) |  |
| Smoking (%) |  |  | <0.001 |
| Uncertain/unknown | 142 (7.09%) | 33 (11.9%) |  |
| No | 1107 (55.2%) | 169 (61.0%) |  |
| Yes | 755 (37.7%) | 75 (27.1%) |  |
| Received flu shot in the last 12 months (%) |  |  | 0.001 |
| Uncertain/unknown | 22 (1.10%) | 12 (4.33%) |  |
| No | 1363 (68.0%) | 181 (65.3%) |  |
| Yes | 619 (30.9%) | 84 (30.3%) |  |
| Received antipneumococcic vaccine at any given time (%) |  |  | 0.005 |
| Uncertain/unknown | 20 (1.00%) | 10 (3.61%) |  |
| No | 1636 (81.6%) | 216 (78.0%) |  |
| Yes | 348 (17.4%) | 51 (18.4%) |  |
