# Supplementary material for: Performance of Machine Learning Algorithms for Predicting Adverse Outcomes in Community-Acquired Pneumonia
Source: Front Bioeng Biotechnol. 2022 Jun 29;10:903426. doi: 10.3389/fbioe.2022.903426 (PMC9278327; doi:10.3389/fbioe.2022.903426)
Supplement: Supplementary file 3 [file Table3.DOCX]

Table S3. Patient characteristics according to one-year post enrollment status stratification.

|  | One year post enrollment status | | p.overall |
| --- | --- | --- | --- |
|  | No | Yes |  |
|  | N=537 | N=1621 |  |
| Cough (%) |  |  | 0.309 |
| No | 30 (5.59%) | 113 (6.97%) |  |
| Yes | 507 (94.4%) | 1508 (93.0%) |  |
| Dyspnea, tachypnoea or hypoxemia (%) |  |  | <0.001 |
| No | 72 (13.4%) | 435 (26.8%) |  |
| Yes | 465 (86.6%) | 1186 (73.2%) |  |
| Fever or hypothermia (%) |  |  | 0.003 |
| No | 224 (41.7%) | 558 (34.4%) |  |
| Yes | 313 (58.3%) | 1063 (65.6%) |  |
| Age (year) (mean (SD)) | 75.9 (15.1) | 59.3 (19.4) | <0.001 |
| Respiratory frequency (/min) (mean (SD)) | 28.6 (16.7) | 28.3 (20.9) | 0.737 |
| Heart rate (/min) (mean (SD)) | 92.5 (17.6) | 90.9 (15.4) | 0.07 |
| SBP (mmHg) (mean (SD)) | 117 (27.2) | 120 (22.1) | 0.008 |
| DBP (mmHg) (mean (SD)) | 70.8 (15.8) | 74.4 (15.9) | <0.001 |
| CURB-65 (mean (SD)) | 2.61 (0.86) | 2.16 (0.59) | <0.001 |
| Hematocrit values (%) (mean (SD)) | 34.8 (6.78) | 38.0 (6.87) | <0.001 |
| Hemoglobin values (g/dl) (mean (SD)) | 12.0 (6.56) | 12.9 (6.24) | 0.003 |
| Leukocytes values (10^9/L) (mean (SD)) | 12.8 (7.66) | 15.4 (80.4) | 0.199 |
| Segmented neutrophils values (%) (mean (SD)) | 80.2 (11.9) | 78.3 (10.6) | 0.001 |
| Platelet values (10^9/L) (mean (SD)) | 420 (320) | 1110 (21572) | 0.199 |
| Creatinine (mg/dL) (mean (SD)) | 1.53 (1.34) | 1.63 (4.20) | 0.41 |
| BUN (mg/dL) (mean (SD)) | 63.2 (42.2) | 49.7 (25.1) | <0.001 |
| Glucose (mg/dL) (mean (SD)) | 137 (62.2) | 137 (57.2) | 0.777 |
| COPD (%) |  |  | 0.634 |
| Uncertain/unknown | 12 (2.23%) | 45 (2.78%) |  |
| No | 440 (81.9%) | 1340 (82.7%) |  |
| Yes | 85 (15.8%) | 236 (14.6%) |  |
| Heart disease (%) |  |  | <0.001 |
| Uncertain/unknown | 6 (1.12%) | 17 (1.05%) |  |
| No | 229 (42.6%) | 956 (59.0%) |  |
| Yes | 302 (56.2%) | 648 (40.0%) |  |
| Diabetes (%) |  |  | 0.416 |
| Uncertain/unknown | 2 (0.37%) | 14 (0.86%) |  |
| No | 444 (82.7%) | 1356 (83.7%) |  |
| Yes | 91 (16.9%) | 251 (15.5%) |  |
| Immunosuppression (%) |  |  | 0.226 |
| Uncertain/unknown | 5 (0.93%) | 7 (0.43%) |  |
| No | 494 (92.0%) | 1517 (93.6%) |  |
| Yes | 38 (7.08%) | 97 (5.98%) |  |
| Malignancy (%) |  |  | <0.001 |
| Uncertain/unknown | 3 (0.56%) | 9 (0.56%) |  |
| No | 463 (86.2%) | 1569 (96.8%) |  |
| Yes | 71 (13.2%) | 43 (2.65%) |  |
| CBVD (%) |  |  | <0.001 |
| Uncertain/unknown | 8 (1.49%) | 6 (0.37%) |  |
| No | 447 (83.2%) | 1545 (95.3%) |  |
| Yes | 82 (15.3%) | 70 (4.32%) |  |
| Kidney disease (%) |  |  | <0.001 |
| Uncertain/unknown | 1 (0.19%) | 13 (0.80%) |  |
| No | 474 (88.3%) | 1519 (93.7%) |  |
| Yes | 62 (11.5%) | 89 (5.49%) |  |
| Liver disease (%) |  |  | 0.228 |
| Uncertain/unknown | 0 (0.00%) | 9 (0.56%) |  |
| No | 522 (97.2%) | 1570 (96.9%) |  |
| Yes | 15 (2.79%) | 42 (2.59%) |  |
| Intravenous drug use (%) |  |  | 0.902 |
| Uncertain/unknown | 2 (0.37%) | 6 (0.37%) |  |
| No | 533 (99.3%) | 1610 (99.3%) |  |
| Yes | 2 (0.37%) | 5 (0.31%) |  |
| Alcoholism (%) |  |  | 0.182 |
| Uncertain/unknown | 7 (1.30%) | 20 (1.23%) |  |
| No | 490 (91.2%) | 1515 (93.5%) |  |
| Yes | 40 (7.45%) | 86 (5.31%) |  |
| Neurological psychiatric disorder (%) |  |  | <0.001 |
| Uncertain/unknown | 15 (2.79%) | 16 (0.99%) |  |
| No | 363 (67.6%) | 1433 (88.4%) |  |
| Yes | 159 (29.6%) | 172 (10.6%) |  |
| Suspected aspiration (%) |  |  | <0.001 |
| Uncertain/unknown | 13 (2.42%) | 7 (0.43%) |  |
| No | 490 (91.2%) | 1591 (98.1%) |  |
| Yes | 34 (6.33%) | 23 (1.42%) |  |
| Hospitalization due to CAP in previous year (%) |  |  | <0.001 |
| Uncertain/unknown | 5 (0.93%) | 4 (0.25%) |  |
| No | 435 (81.0%) | 1431 (88.3%) |  |
| Yes | 97 (18.1%) | 186 (11.5%) |  |
| Overcrowding (%) |  |  | 0.579 |
| Uncertain/unknown | 10 (1.86%) | 28 (1.73%) |  |
| No | 514 (95.7%) | 1565 (96.5%) |  |
| Yes | 13 (2.42%) | 28 (1.73%) |  |
| Smoking (%) |  |  | <0.001 |
| Uncertain/unknown | 56 (10.4%) | 117 (7.22%) |  |
| No | 320 (59.6%) | 875 (54.0%) |  |
| Yes | 161 (30.0%) | 629 (38.8%) |  |
| Received flu shot in the last 12 months (%) |  |  | <0.001 |
| Uncertain/unknown | 18 (3.35%) | 16 (0.99%) |  |
| No | 346 (64.4%) | 1117 (68.9%) |  |
| Yes | 173 (32.2%) | 488 (30.1%) |  |
| Received antipneumococcic vaccine at any given time (%) |  |  | 0.002 |
| Uncertain/unknown | 15 (2.79%) | 15 (0.93%) |  |
| No | 419 (78.0%) | 1334 (82.3%) |  |
| Yes | 103 (19.2%) | 272 (16.8%) |  |
